# Comparative Research of Chemical Profiling in Different Parts of *Fissistigma oldhamii* by Ultra-High-Performance Liquid Chromatography Coupled with Hybrid Quadrupole-Orbitrap Mass Spectrometry

**DOI:** 10.3390/molecules26040960

**Published:** 2021-02-11

**Authors:** Haibo Hu, Yau Lee-Fong, Jinnian Peng, Bin Hu, Jialin Li, Yaoli Li, Hao Huang

**Affiliations:** 1 National Engineering Research Center for Modernization of Traditional Chinese Medicine—Hakka Medical Resources Branch, School of Pharmacy, Gannan Medical University, Ganzhou 341000, China; planthunter@outlook.com (H.H.); pengjinnian2017@gmu.edu.cn (J.P.); hubin12332117@sina.com (B.H.); jialinli2005@gmu.edu.cn (J.L.); 2 Department of Biology, Animal Physiology and Neurobiology Section, Katholieke Universiteit Leuven, Naamsestraat 59, Box 2465, 3000 Leuven, Belgium; 3 State Key Laboratory of Quality of Traditional Chinese Medicine, Macao University of Science and Technology, Macau 999078, China; lfyau@must.edu.mo; 4 School of Pharmaceutical Sciences, Peking University, Beijing 100191, China

**Keywords:** *Fissistigma oldhamii*, aristolactam, Q-Exactive, MS fragmenter, XCMS

## Abstract

The roots of *Fissistigma oldhamii* (FO) are widely used as medicine with the effect of dispelling wind and dampness, promoting blood circulation and relieving pains, and its fruits are considered delicious. However, Hakka people always utilize its above-ground parts as a famous folk medicine, Xiangteng, with significant differences from literatures. Studies of chemical composition showed there were multiple aristolactams that possessed high nephrotoxicity, pending evaluation research about their distribution in FO. In this study, a sensitive, selective, rapid and reliable method was established to comparatively perform qualitative and semi-quantitative analysis of the constituents in roots, stems, leaves, fruits and insect galls, using an Ultra-High-Performance Liquid Chromatography coupled with Hybrid Quadrupole Orbitrap Mass Spectrometry (UPLC-Q-Exactive Orbitrap MS, or Q-Exactive for short). To make more accurate identification and comparison of FO chemicals, all MS data were aligned and screened by XCMS, then their structures were elucidated according to MS^n^ ion fragments between the detected and standards, published ones or these generated by MS fragmenter. A total of 79 compounds were identified, including 33 alkaloids, 29 flavonoids, 11 phenylpropanoids, etc. There were 54 common components in all five parts, while another 25 components were just detected in some parts. Six toxic aristolactams were detected in this experiment, including aristolactam AII, AIIIa, BII, BIII, FI and FII, of which the relative contents in above-ground stems were much higher than roots. Meanwhile, multivariate statistical analysis was performed and showed significant differences both in type and content of the ingredients within all FO parts. The results implied that above-ground FO parts should be carefully valued for oral administration and eating fruits. This study demonstrated that the high-resolution mass spectrometry coupled with multivariate statistical methods was a powerful tool in compound analysis of complicated herbal extracts, and the results provide the basis for its further application, scientific development of quality standard and utilization.

## 1. Introduction

*Fissistigma oldhamii* (Hemsl.) Merr. (FO), a medical herb from southern China, named ‘Guangxiangteng,’ ‘Xiangteng,’ ‘Zuandifeng,’ or ‘Tenglongyan,’ is a perennial woody climbing vine of the family Annonaceae [1] and was first recorded as Guangxiangteng in *Zhiwu Mingshi Tukao* (AD1848), where it was said that “it was produced in Nan’an (Dayu Ridge in Ganzhou, China)” [2]. Its dried root was commonly used with the effects of dispelling wind and dampness, promoting blood circulation and relieving pains, and the mature fruits were sweet and edible, called “fox peach” in Longnan, Ganzhou, China [1,3,4]. However, in the Hakka areas, its above-ground parts, including leaves and stems sometimes mixed with insect gall, were widely used for treating gynecology inflammation, expelling wind and dampness, and was called as ‘Xiangteng’ due to its aromatic smell [5]. Moreover, in Dong medicine, its stems and leaves were also used for treating fractures and edema, and the whole plants were utilized in Yao medicine to cure rheumatism, bone pain, numbness of hands and feet, sequelae of polio and children’s convulsions [6]. Hence, individual parts of FO displayed different medicinal usages as well as different pharmacological activities, which might be relevant to their chemical differences.

Chemical differences among individual parts of plant materials are of great significance for their pharmacological activities and safety, which are also critical for their authentication and quality evaluation [7,8]. Studies showed that volatile oil, alkaloids, flavonoids, steroids and organic acids were the major constituents of FO that exhibited anti-tumor, anti-inflammatory, analgesic, smooth muscle relaxation and other biological activities. Remarkably, FO also contained the aristolactam alkaloids that possessed strong nephrotoxicity [9,10,11,12]. However, comprehensive studies investigating the chemical differences among individual parts of FO are still scarce. In recent years, the advanced developed quadrupole-electrostatic field orbitrap high-resolution mass spectrometer (UPLC-Q-Exactive Orbitrap MS), which combines quadrupole ion selection and Orbitrap high-resolution scanning, was capable of conducting high-throughput trace target or non-target screening, and has become a highly reliable technique for qualitative and quantitative analysis of multiple components in complex [13]. For instance, in omics researches it has been applied and widely used in the component analysis of complex systems, such as food, natural products and traditional Chinese medicine (TCM) [14,15,16,17].

However, the related references for the identification of the minor constituents in TCM are not abundant [18,19]. Further, the MS identifications normally were performed via the MS^n^ comparison with database, standards, or published spectra [20,21], while the interpretation of an “unknown” is a crucial task difficult to tackle, due to lack of authentic standards and scarce information available (spectra or fragmentation) [22]. Hence, we herein carried out an advanced UPLC-Q-Exactive Orbitrap MS approach to establish a rapid and reliable method, in which all MS data were aligned and screened by XCMS (or XCMS Online, a web-based platform to process metabolomic data) [23], then their structures were elucidated according to MS^n^ ion fragments between the detected and several standards, in a manually built database. Also, for these “unknown” ones, a fragmentation prediction program (MS fragmenter) was used to evaluate the chemical structures and predict mass spectral fragments based on the cleavage rules [24]. Meanwhile, the multivariate statistical analysis was subsequently applied for chemical content discrimination of different FO parts. Via qualitative and quantitative research of the biological active and toxic FO compounds, this study is to provide chemical evidence for their differentiated efficacy and usage safety, including roots, stems, leaves, fruits and insect galls. This study exhibited a rapid, sensitive, selective and reliable method to immensely eliminate the single-data error and comprehensively interpret the chemical basis for different medicinal parts of herbs. The results will provide a basis for further studies of FO, such as the formulation and practical application of its quality standards.

## 2. Results and Discussion

### 2.1. Optimization of UPLC Q-Exactive Orbitrap MS

Mobile phases provided different separation conditions, in which the buffer salt played one of the most important roles to affect the ionization of target compounds at ESI source [16,25,26], resulting in reducing or increasing sensitivity of FO components detected by mass spectrometry. Hence, four systems were tested in our experiments, including pure water, 0.1% formic acid, 5 mM ammonium acetate, and 0.1% formic acid plus 5 mM ammonium acetate. The results showed 0.1% formic acid plus 5 mM ammonium acetate were selected due to better separation and higher sensitivity for FO extracts. Furthermore, full MS and full MS/dd-MS2 mode were generally used to capture all the targeted range of MS^1^ and MS^1,2^ fragmentation information for qualitative analysis and quantitative comparison. Full MS/dd-MS^2^ mode always provided more information for targeted or unknown compound identification [27]; however, more scanning time was also required for monitoring MS^2^ ions, and therefore reduced the sensitivity. Hence, full MS/dd-MS^2^-TOP 5 (the top 5 ions’ MS^2^) was selected to detect FO components both in negative and positive mode with the range of *m*/*z* 50–1000. The high-resolution mass spectrometry (HRMS) always has two key parameters as resolution and extracted mass tolerance, which affected a lot for qualitative and quantitative detection [16]. Hence, in this study we evaluated the tolerance of 2–20 µg mL^−1^ (or ppm; 1 ppm is approximately equal to 1 µg mL^−1^ in water) and resolution of 17,500 to 140,000, and finally 5 µg mL^−1^ tolerance with 70,000 resolution were used for this experiment. Compared with TOF or triple MS, the Orbitrap HRMS was proven to have higher sensitivity in full scan mode, higher sensitivity and intensity range [28], allowing a high-accuracy within 5 ppm [29]; hence, the optimization in this study reached the excellent level of this powerful technique.

### 2.2. Total Ion Chromatogram Comparison and Qualitative Analysis of FO Parts

**Figure 1:**
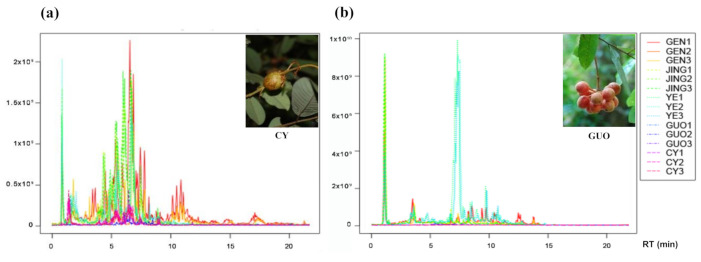
Total ion chromatograms (TICs) of different parts of *F. oldhamii* under positive (**a**) and negative (**b**) ion mode. GEN1–3: roots; JING1–3: stems; YE1–3: leaves; GUO1–3: fruits; CY1–3: insect galls.

The chemical constituents are affected by various factors such as the origin place, the harvesting season and the growing year [30]. To evaluate the variation of FO inherently associated with the different parts, we specially collected diverse whole herbs with fruits from the first literature-recorded place (Ganzhou, China) for the comparative study. Based on the above experimental conditions, a total of 15 samples of various FO parts were detected by HRMS, and all these ion chromatograms (TIC) in both positive ion and negative ion modes were obtained respectively. The chromatograms of all FO samples were compared by XCMS [31,32,33] after subtracting the background with blank samples under Xcalibur, and are shown in Figure 1. The responses of roots, stems, leaves and insect galls in positive ion mode were relatively strong, while the leaves and stems highly responded in negative ion mode, indicating obvious differences of the types or relative contents of the compounds in FO parts. A total of 79 components were identified, including 33 alkaloids, 29 flavonoids, 11 phenylpropanoids, 4 sesquiterpene and triterpenes, 1 quinone and 1 phenolic acid component (Table 1 and Table 2). Among them, six kinds of aristolactams were detected, namely, Aristolactam A II, A III a, BII, BIII, F I and F II.

### 2.3. Different Components among FO Parts Screened by XCMS

All the data were processed by XCMS with sample name, accurate mass-to-charge ratio, retention time, *p* value, Q value and intensity of each fragment. The zero-intensity fragments were manually searched and obtained, which meant the components represented by these ions were not detected or distributed in the related samples. Moreover, only the ones that showed zero value in all three-parallel samples of each part were considered as the none-distributed components. In this experiment, XCMS-aligned data contained 12,238 fragments in positive-ion- mode data and 19,861 in negative-ion-mode data. After rearranging the retention times in Excel, the zero-intensity fragments at the same or very-close (<0.05) retention time were used for qualitative analysis. A total of 25 differential components of FO parts were identified and obtained, including 6 alkaloids, 13 flavonoids, 3 phenylpropanoids, 3 triterpenes (Table 2).

The result indicated an interesting phenomenon about the chemical absence in plant part(s). For instance, three components were only detected in roots, such as norfissilandione, norcepharadione B and gedunin. This may function specially for roots, generated by specific root tissues (e.g., root tip, root hairs, etc.) or the different environments (lightless in soil) from other parts [34,35], pending further research, like laser-capturing to detect the tissue or cell chemicals [36,37]. In contrast, there were five components distributed in all the parts except root, including artabotryside A, apiin, nicotiflorin, eupatolin and quercetin-3-O-rhamn-oside, which may be generated by the above-ground tissues. Meanwhile, the stem and insect gall showed same chemical distribution, and two compounds, claussequinone and methyl 3-(2-oxo-2-prop-2-enoxyethyl)-1-benzofuran-2-carboxylate, were only absent in these two parts. Leaves and fruits had the similar results that cnidimol B, claussequinone, morusin and fissohamione distributed only here, while aristolactam FI was absented. Aristolactam FII, Kwangsienin A and Isopedicin were only absent in leaves, while isoquercitrin was just detected in leaves. The components of these different distributions were determined by the specific metabolic functions of these tissues, or the different external environment leading to plant defense [38] or other actions, which benefited the metabolic mechanism research [39]. In other aspects, the identification of Chinese traditional patent medicine is more difficult than that of Chinese herbal medicine [40], due to the usage of their powders or extracts. Although the identification techniques (liquid or gas chromatography, etc.) have been developed significantly already, it is still hard to find a specific way to tell the real resource. Hence, these differences of FO compounds can be used as identification markers of which FO tissues used in proprietary Chinese medicine, increasing the safety and efficacy of medication.

### 2.4. Multivariate Statistical Analysis and Comparison

**Figure 2:**
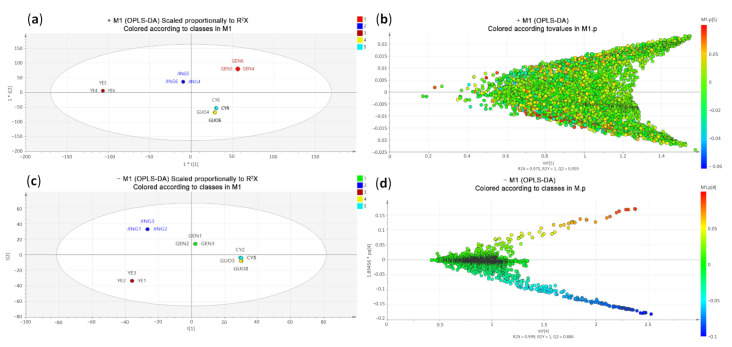
OPLS-DA scores plots and loading plots of 15 samples of different parts of *F. oldhamii*. (**a**–**d**) are positive and negative ion mode data separately.

In recent years, high-resolution LC/MS technology combined with multivariate statistical methods were applied to accurately perform for the omics study of traditional Chinese medicine [41,42,43]. In this study, we also performed this method to compare the component analysis of multiple samples. After sieving by XCMS, all the data without zero were considered as the common fragments of each FO part. Multivariate statistical analysis was performed to compare and analyze their differences, including principal component analysis (PCA), partial least-squares discrimination analysis (PLS-DA) and orthogonal partial least-squares discrimination analysis (OPLS-DA). The purpose was to establish a relationship model between component expression and samples to realize the prediction and judgment analysis of the data category. The steps included importing the excel data into the SIMCA-P 14.0 for modeling and automatically fitting to screen the different models [44,45,46,47,48]. The experimental data was optimally modeled under OPLS-DA, and the UV model was built with the following parameters: positive ion mode: R^2^X = 0.975, R^2^Y = 1, Q^2^ = 0.959; negative ion mode: R2X = 0.999, R2Y = 1, Q2 = 0.886. The closer R^2^ and Q^2^ values were to 1, the better the model was [47,48]. From Figure 2a,b, the OPLS-DA model of positive and negative ion data has been successfully established, and all the FO samples were clearly clustered into five categories, indicating that the expressions of common components in FO parts were significantly different, and the fruit and insect galls showed much similarity under this model.

**Figure 3:**
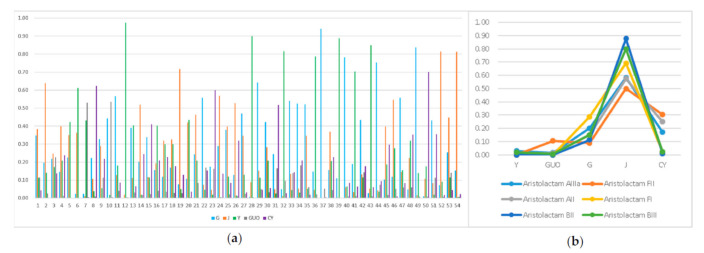
The relative percentage of 54 compounds (**a**) and 6 aristolactams (**b**) in different parts of *F. oldhamii*. GEN1–3: roots; JING1–3: stems; YE1–3: leaves; GUO1–3: fruits; CY1–3: insect galls; 1–54: compounds in Table 1.

Figure 2c,d showed the loading plots of all the fragments in 15 samples, in which the farther the spots away from the center, the greater the component difference in FO parts. The extent of component differences was evaluated by VIP values (variable importance for the projection importance) [44,45] to compare the strength and explanatory power of each component expression on their classification and discrimination. The ions with VIP value > 1.0 were consider as the fragments of markers, and their expression intensities were used to compare relative content in different FO components. The fragments farther from the clusters had higher VIP values, and all the ions with a VIP value greater than 1 were screened out in Excel format. According to the retention time and mass-to-charge ratio of these ions, the MS data were targeted for searching, and the corresponding common components were sorted out. Afterwards, the average intensity of each compounds in three parallel data for each FO part was used as the comparison benchmark for relative content (%) [30,47,48] (Figure 3). A total of 54 components (markers) in different FO parts were obtained and identified, including 27 alkaloids, 16 flavonoids, 8 phenylpropanoids, 1 sesquiterpene, 1 quinone and 1 phenolic acid component (see Table 1).

### 2.5. Semi-Quantitative Analysis

Figure 3 showed the relative percentage of 54 common components, in which the content of most compounds in roots, stems and leaves were generally higher than others. Their intensities were used to express the relative percentage content in Figure 3a,b. Comparing the contents in FO parts, most components were significantly more in roots, stems and leaves than other parts. The constituents mainly distributed in FO roots were glaucine, aloenin, duguevanine, licocoumarin A, clausamine F, thunberginol C, oxocrebanine, anolobine, etc., of which, notably, isoquercitrin, norfissilandione, norcepharadione B and gedunin were only detected in roots. The constituents dominating in stems were found to be glucosyringic acid, dehydrodeguelin, lactucin, etc., which, being polar species, can be transported in stem xylems [49]. The main components detected in leaves were astilbin, corytuberine, demethylmoracin I, calycinine, noraristolodione, ammidin, 5, 6, 7, 8, -Tetramethoxyflavone, Aristolactam BIII, etc., while fruits had the biggest relevant contents of fissistigmatin A, xylopine and fissilandione. However, only crebanine was mainly distributed in insect galls, and with good antibacterial activity [50,51]; crebanine should be a way to protect the plant from insect injured.

### 2.6. Aristolactams Differences

Since the first-reported over 100 cases of aristolochic acid nephropathy (AAN) in Belgium [52], much attention has been paid to the distribution and toxicity of aristolochic acid (AA) and aristolactam (AL) in traditional herbs, especially for the traditional Chinese medicine (TCM) [53]. Epidemiological studies showed that AA and AL exposure is associated with a high risk of nephrotoxicity and upper urinary tract carcinoma (UUC), indicating that these toxic compounds’ distribution was of wide concern [54,55,56,57,58]. According to the previous studies, 14 aristolactams have been isolated or detected from FO, including aristolactam A II, A III a, B II, B III, F I, F II, GI, GII, oldhamactam, stigmalactam, piperolactam C, enterocarpam I, velutinam and goniothalactam, most of which were derived from FO stems [9,59,60,61].

In this study, a total of six aristolactams were identified in different parts of FO, including Aristolactam AII, AIIIa, BII, BIII, FI and FII. After the XCMS screening, three differently distributed aristolactams were found, in that aristolactam B II and F I were not found both in leaves and fruits and aristolactam F II only not in leaves (Figure 3b, Table 2). For all six aristolactams, they were mainly distributed in stems, insect galls and roots. Notably, all the relative content of aristolactams in above-ground stems were more than 50%, quite higher than other parts. For example, the percentages of aristolactam BII and BIII in stems were even 88% and 84%, respectively. FO leaves and fruits had less aristolactams both in number and content. However, eating fruits was considered as a risk due to these toxic components, and both underground roots (Guangxiangteng) and above-ground stems and leaves (Xiangteng) should also be carefully used as medicine, especially for oral administration.

### 2.7. Identification and Component Pyrolysis Based on MS Fragmentater

**Figure 4:**
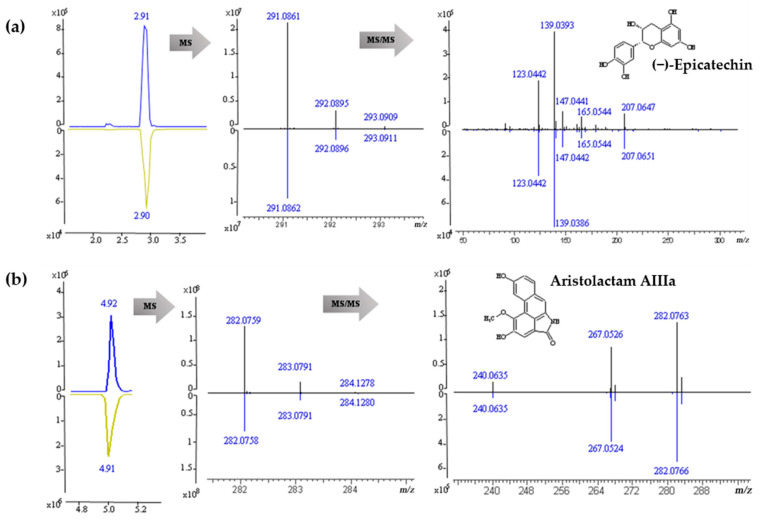
MS^1^ (**a**) and MS^2^ (**b**) spectra of (−)-epicatechin and aristolactam AIIIa (upper panel: sample, lower panel: standard).

**Figure 5:**
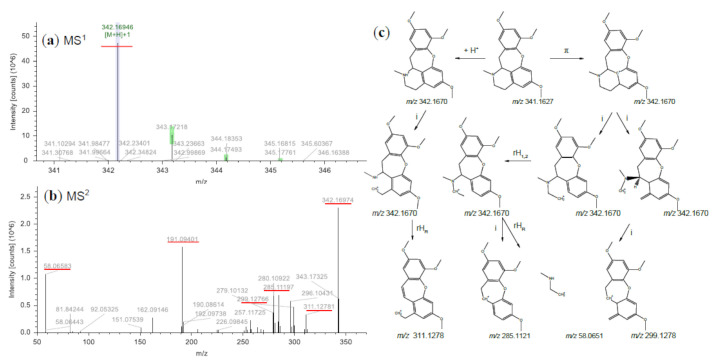
MS^1^ (**a**) and MS^2^ (**b**) spectra of Fissistigine C (*m*/*z* 342.1694 [C_20_H_24_NO_4_]^+^) and its main mass fragmentation pathway (**c**) under positive ion mode. π: π-bond dissociation, i: inductive cleavage, +H^+^: protonation, rHR: charge-romate rearrangement, rH_1,2_: 1,2 Hydrogen rearrangements.

For component identification, the published spectra under same MS condition were quite useful to confirm the structures with the comparison of the experimental spectra, especially for the MS^2^ spectra. However, most FO compounds did not have these sources in public databases. Hence, several standard components were used to assist the identification, such as (−)-epicatechin and aristolactam AIIIa shown in Figure 4. Meanwhile, to interpret other components without standards or published spectra, their MS fragmentations were predicted by MS Fragmentater [62,63] for the comparison with the detected fragments. Taking the cleavage of Fissistigine C [64] as an example, it was detected in positive ion mode with a strong molecular ion peak, *m*/*z* 342.1699[C_20_H_24_NO_4_]^+^. Its fragmentation under MS Fragmentater mainly included the cleavage of nitrogen-containing heterocycles and oxygen-containing 7-membered rings. The MS fragments included *m*/*z* 342.1670 [C_20_H_24_NO_4_]^+^, *m*/*z* 314.1387 [C_18_H_20_NO_4_]^+^, *m*/*z* 313.1434 [C_19_H_21_O_4_]^+^, *m*/*z* 311.1278 [C_19_H_19_O_4_]^+^, *m*/*z* 310.1438 [C_19_H_20_NO_3_]^+^, *m*/*z* 299.1278 [C_18_H_19_O_4_]^+^, *m*/*z* 285.1121 [C_17_H_17_O_4_]^+^, *m*/*z* 283.0965 [C_17_H_15_O_4_]^+^, *m*/*z* 204.1019 [C_12_H_14_NO_2_]^+^, *m*/*z* 191.0940 [C_11_H_14_NO_2_]^+^, *m*/*z* 176.1070 [C_11_H_14_NO]^+^, *m*/*z* 151.0754 [C_9_H_11_O_2_]^+^, *m*/*z* 148.0757 [C_9_H_10_NO]^+^, *m*/*z* 137.0597 [C_8_H_9_O_2_]^+^, *m*/*z* 133.0648 [C_9_H_9_O]^+^, *m*/*z* 58.0651 [C_3_H_8_N]^+^, *m*/*z* 56.0495 [C_3_H_6_N]^+^, *m*/*z* 44.0495 [C_2_H_6_N]^+^ and *m*/*z* 42.0338 [C_2_H_4_N] ^+^, etc. Figure 5a,b shows MS scan results, exhibiting strong ions of *m*/*z* 342.1697 [C_20_H_24_NO_4_]^+^, *m*/*z* 191.0940 [C_11_H_14_NO_2_]^+^ and *m*/*z* 58.0658 [C_3_H_8_N]^+^, and other obvious ones of *m*/*z* 311.1278 [C_19_H_19_O_4_]^+^, *m*/*z* 299.1277 [C_18_H_19_O_4_]^+^ and *m*/*z* 285.1120 [C_17_H_17_O_4_]^+^. The experimental fragmentation fit very well with the fragments predicted above; therefore, it was identified as Fissistigine C. The fragment ions were generated via several processes, including protonation, π bond dissociation, inductive cleavage, rearrangements, etc. (Figure 5c).

### 2.8. Method Validation

Based on the above optimized condition, the mixed stock solution was diluted with methanol to different concentrations for constructing calibration curves. The ratio of peak areas (y) of each compound was plotted against the concentrations (x, ng mL^−1^). All the curves exhibited good linearity with determination coefficients (R^2^) from 0.9988 to 0.9999. The LOQ and LOD were evaluated by the signal-to-noise (S/N) of 10 and 3. The values of LOD and LOQ in this experiment were 0.12–6.41 and 0.21–5.45 ng mL^−1^, respectively.

Meanwhile, the intra- and inter-day precision were calculated by analyzing the stock solution under the above optimized conditions, with respective RSD values less than 4.8% and 4.9%. In addition, the acceptable RSD values of repeatability (*n* = 6) and stability in 8 h (*n* = 6) were 2.3–4.5% and 2.8–4.8%.

The recovery was validated by adding a known amount of stock standard at different concentration levels (high, middle and low, *n* = 3) into a selected sample. The mixtures were analyzed in triplicate with the optimized method. The recovery of this method varied from 99.5% to 103.5% with the RSD value between 0.5% and 3.58%. The detailed data are listed in Table 3.

## 3. Materials and Methods

### 3.1. Reagents and Materials

Eight standard compounds were purchased from Yuanye Biotechnology Co., Ltd. (Shanghai, China), including syringic acid (LOT: S23F7K9842), (−)-epicatechin (LOT: C16N6Q5881)**,** crebanine (LOT: R15D8F50600), corytuberine (LOT: Z11M8S35795), isopedicin (LOT: X12D8L50492), aristolactam BII (LOT: X12D8L50494), aristolactam F I (LOT: X12D8L50490) and aristolactam AIIIa (LOT: X12D8L50493). The purity of each chemical was above 95%. LC/MS grade methanol, formic acid, acetonitrile, and ammonium acetate were purchased from Fisher Scientific (Fair Lawn, NJ, USA), and water was obtained from a Milli-Q water purification system (Millipore, Bedford, MA, USA).

With accordance to the first recorded place, Nan’an (Ganzhou, China) [2], the plant materials, ten adult herbs with fruits, were collected as samples from similar sites in Jiulian mountain national forest in Jiulian Mountain, Longnan County, Ganzhou City, China, including Jiulianshan forestry station (24°62′ N, 114°56′ E), Aobei (24°61′ N, 114°55′ E), Shuangdun (24°62′ N, 114°54′ E), Jiulianshan reserve management station (24°46′ N, 114°54′ E) and Huangniushi Linchang (24°50′ N, 114°41′ E). All the herbs were segmented around 20–30 cm long, and then kept in clean cartons under −20 °C for further experiments. All the samples were identified as *Fissistigma oldhamii* (Hemsl.) Merr. by Professor Jialin Li and Professor Haibo Hu of the School of Pharmacy, Ganzhou Medical University. A voucher specimen was kept in the Herbarium of Chinese Medicine of Gannan Medical University (No. XT-CY1501).

### 3.2. Preparation of Standard and Sample Solution

In order to eliminate the possible errors of single detection, each sample was prepared in three parallel groups. For each group, 10 FO plants were cleaned and separated into the roots, stems, leaves, fruits and insect galls. These different parts were dried under 45 °C for 48 h, then smashed into powder. A 40-mesh pharmacopoeia sieve was used to obtain average particles, in which 1 g of each part were sampled and put in a closed centrifuge tube with 20 mL methanol. The mixtures were steeped for 1 h and extracted ultrasonically for 3 times, lasting 0.5 h each time with an interval of 4 h. After centrifugation (3000 rpm, 10 min), the supernatant was taken, diluted by 10-fold for HRMS. The samples of each part were made by the above same method for 3 times and named GEN1–3, JING1–3, YE1–3, GUO1–3, CY1–3, indicating roots, stems, leaves, fruits and insect galls, respectively. The stock solutions of standard compounds in methanol were prepared in 4 μg·mL^−1^ of each compound and also a mixed solution. All samples and standard solution were stored at 4 °C and be filtered through a 0.2 μm filter before analysis.

### 3.3. UHPLC and MS/MS Conditions

The ultraperformance liquid chromatography-tandem mass spectrometry were performed using an Ultimate 3000 UHPLC system with a WPS-3000 auto-sampler, coupled to a Q-Exactive Qibitrap MS spectrometer, which is combined with quadrupole ion selection and Orbitrap high-resolution scanning (Thermo Fisher Scientific, Waltham, MA, USA). The chromatographic separation was carried out on a Waters Acquity UPLC BEH C18 column (100 mm × 2.1 mm, 1.7 μm particle size; Waters, Milford, CT, USA). With 0.3 mL·min^−1^ flow rate, the column was set at 35 °C and the injection volume was 10 μL. The mobile phase was composed of acetonitrile (eluent A), water containing 0.1% formic acid and 5 mM ammonium acetate (eluent B). The gradient elution conditions were set as follows: 0–3 min, 12–30% A; 3–5 min, 30–50% A; 5–10 min, 5%0–95% A; 10–15 min, 95% A; 15–20 min, 95–100% A; 20–22 min, 100% A; in 22.01 min, switched to 12% A and kept up to 5 min to equilibrate the system.

For MS detecting, a Q-Exactive-Qibitrap-MS spectrometer was fitted with a heated electrospray ionization (ESI) ion source in both negative and positive ionization mode at full scan mode ranged *m*/*z* 100–1000. To aid the structural identification of the components, top 5 ions’ MS/MS fragmentation (dd-MS^2^-TOP 5) was operated with the range of *m*/*z* 50–1000. The optimal MS parameters were as follows: spray voltage −2.8 Kv/+3.5 Kv; sheath gas flow rate, 35 arbitrary units; auxiliary gas flow rate, 10 arbitrary units; capillary temperature, 320 °C; auxiliary gas heater temperature, 350 °C. The resolution of full scan and dd-ms^2^ were 70,000 and 35,000 FWHM (full width at half maximum), while their AGC target were set as 3 × 10^6^ and 1 × 10^5^, with their maximum IT (the maximum injection time allowed to obtain the set AGC target) 100 and 50 ms, respectively. The stepped NCE (normalized collision energy) was set to 35 V for MS/MS acquisition.

### 3.4. Data Processing and Analysis

All the data operation, acquisition and analysis were done by Xcalibur 4.2 (Thermo Fisher Scientific Inc., Waltham, MA, USA), ACD/MS Workbook Suite 2020 (Advanced Chemistry Development, Inc. Toronto, ON, Canada), combined with MS Fragmentater 2020 (ACD/labs), XCMS-online (Scripps Research Institute, La Jolla, CA, USA) and SIMCA 14.0 (Umetrics, Umeå, Sweden). The original UHPLC-MS data of each samples were exported, and their background were subtracted from mass spectrum data of the blank solvent using Xcalibur for both positive and negative ion data. Then, the processed data were imported to XCMS to extract peaks and align chromatograms to make the comparison more accurate than manual work. The processing parameters were as follows: mass range: 100–1000 Da; mass tolerance: 10 ppm; RT tolerance [min]: 0.05; S/N threshold: 5. Then, all the aligned MS data of FO samples without interfering peaks were obtained, including the retention time and precise molecular mass which were necessary for chemical identification.

Before detection, the published compounds of FO were comprehensively collected by checking Dictionary of Natural Products, Sci Finder, Web of Science, CNKI and other databases. Then, a manual compound library was created, including the chemical name, molecular formula, exact molecular weight, and structure information. Under Xcalibur 4.2, the mass spectrums and their secondary spectrum (top 5 ions of MS^2^ fragmentation) of the molecular ion peaks were screened and checked by comparing the detected formula and MS fragments with the component library we built, and most compounds were targeted and identified. Then, their structures were elucidated by comparing with their detected MS^n^ ion fragments and the fragmentation patterns of each compound were summarized and further verified according to MS Fragmenter.

Meanwhile, the aligned Excel data converted by XCMS were manually screened for the fragments with zero intensity, represented the absence of the corresponding compounds in the related FO part. By this method, the different compounds among FO parts were obtained. Secondly, the data without zero-intensity fragments were carried out with multivariate statistical analysis by SIMCA to identify the difference of component contents in each FO part. PLS-DA and OPLS-DA analysis of the processed data were then performed to identify the compounds with VIP > 1, which implied the markers as the main components with significant content differences among FO parts. Meanwhile, their intensity was considered as the relative content and were used for the content comparison.

## 4. Conclusions

In this paper, a rapid and reliable method using UHPLC-Q -Exactive–MS was established to perform qualitative and semi-quantitative analysis of the constituents in all *Fissistigma oldhamii* (FO) parts (roots, stems, leaves, fruits and insect galls). The analysis revealed a high content of virous compositions in FO, in which alkaloids and flavonoids were the main compounds. Via comparing both MS^1^ and MS^2^ spectra with standards and fragmentations, 79 components were identified, and they were compared by XCMS screening and multivariate statistical analysis. This showed that there were 25 components differentially distributed in FO parts, and 54 common ones (markers) were recognized with obviously different relative content, most of which exhibited quite higher contents in the roots and leaves. The results showed that the chemical components of the roots, stems, leaves, fruits and insect galls had obvious differences in types and relative contents.

Notably, due to the distribution of highly nephrotoxic aristolactams, all FO parts should be carefully valued for oral administration, especially for eating fruits. The method used in our experiment can quickly recognize the component differences between multiple samples, while the MS^n^ comparison based on MS fragmenter can provide much more information for the identification of “unknown” compounds. However, the high-density data and charged software could be a barrier for the method application. In summary, the high-resolution LC/MS technology combined with multivariate statistical methods can accurately perform component analysis of different FO samples, and should be used for studies of other traditional Chinese medicine. Clear distributions of effective or toxic components in plants can provide a basis for the medicinal and edible usage of various plant tissues and for further studies of the metabolic process and mechanism of plant components.

## Figures and Tables

**Table 1:** Common compounds in five parts of *F. oldhamii.*

No.	Name	RT	VIP	Formula	Ion Mode	Observed *m*/*z*	Calculated *m*/*z*	Diff. (ppm)	MS/MS Fragments
**1**	Glucosyringic Acid	1.45	1.32	C_15_H_20_O_10_	[M − H]^−^	359.0986	359.0984	0.64	198.0490, 197.0456, 182.0222, 153.0557, 138.0322, 89.0243
**2**	Dehydrodeguelin	1.86	2.00	C_23_H_20_O_6_	[M + H]^+^	393.1335	393.1333	0.56	393.1335, 377.1386, 351.0865, 175.0752
**3**	**Syringic acid**	2.51	3.07	C_9_H_10_O_5_	[M + H]^+^	199.0599	199.0601	−1.00	171.0650,156.0416,140.0480,125.0223
**4**	**(−)-Epicatechin**	2.90	2.16	C_15_H_14_O_6_	[M + H]^+^	291.0861	291.0863	−0.69	207.0647, 165.0544, 147.0441, 139.0393, 123.0442
**5**	Stigmahamone I	2.98	2.53	C_19_H_20_O_7_	[M − H]^−^	359.1136	359.1136	−0.11	313.0718, 299.0561, 297.0405, 282.0171, 166.9985
**6**	Astilbin	3.08	1.72	C_21_H_22_O_11_	[M − H]^−^	449.1092	449.1089	0.64	449.1080, 343.0826, 303.0804, 299.0926, 285.0421, 151.0032
**7**	Lactucin	3.71	2.53	C_15_H_16_O_5_	[M + H]^+^	277.1063	277.1071	−2.71	259.1221, 245.0456, 173.0611, 99.0087, 91.0572
**8**	*N*-Caffeoyl-*O*-methyltyramine	4.04	1.95	C_18_H_19_NO_4_	[M + H]^+^	314.1384	314.1387	−0.95	297.1176, 265.0858, 237.0908, 233.0597,205.0646
**9**	**Corytuberine**	4.13	2.23	C_19_H_21_NO_4_	[M + H]^+^	328.1539	328.1543	−1.22	297.1118, 282.0884, 265.0856, 237.0907
**10**	4,6-dimethoxy-2,5-quinodihydrochalcone	4.26	2.08	C_17_H_16_O_5_	[M − H]^−^	299.0930	299.0925	1.68	173.0607, 164.0107, 125.0243, 108.0209, 91.0541
**11**	Haplotubinone	4.32	1.24	C_19_H_23_NO_4_	[M + H]^+^	330.1695	330.1700	−1.51	299.1274, 239.1066, 192.1018, 175.0752, 151.0754, 143.0492, 137.0597
**12**	Afzelin	4.91	2.43	C_21_H_20_O_10_	[M − H]^−^	431.0984	431.0984	0.07	431.0984, 399.0730, 371.0767, 341.0667, 311.0562, 283.0614
**13**	(−)-Epicatechin gallate	4.93	1.68	C_22_H_18_O_10_	[M − H]^−^	441.0827	441.0827	−0.05	39.0930, 382.0699, 295.0615, 278.0071, 250.0122, 173.0609
**14**	**Aristolactam AIIIa**	4.94	3.41	C_16_H_11_NO_4_	[M + H]^+^	282.0759	282.0761	−0.71	282.0763, 267.0526, 240.0635
**15**	**Coclaurine**	5.05	1.04	C_17_H_19_NO_3_	[M + H]^+^	286.1435	286.1438	−1.05	267.0527, 239.0581, 211.0629,183.0682
**16**	Isoboldine	5.22	2.43	C_19_H_21_NO_4_	[M + H]^+^	328.1542	328.1543	−0.30	328.1543, 329.1577, 311.1288, 297.1124, 265.0858,237.0910, 192.1023
**17**	Naringin dihydrochalcone	5.36	1.48	C_27_H_34_O_14_	[M − H]^−^	581.1882	581.1876	1.07	301.0312, 300.0278, 274.0806, 273.0772, 167.0349, 125.0244
**18**	Proanthocyanidin A_2_	5.17	2.89	C_30_H_24_O_12_	[M − H]^−^	575.1202	575.1195	1.22	575.1204, 449.0883, 423.0727, 407.0773, 394.0696, 271.0253, 243.0302, 161.0245, 137.0245, 125.0245
**19**	Thaipetaline	5.75	2.67	C_20_H_23_NO_5_	[M + H]^+^	358.1643	358.1649	−1.68	358.1647, 341.1625,311.1277, 299.1299, 192.1012, 74.0606, 60.0442
**20**	Demethylmoracin I	5.83	2.39	C_19_H_18_O_4_	[M + H]^+^	311.1283	311.1278	1.61	296.1047, 295.0962, 280.1093, 265.0857, 237.0909, 219.0796, 92.0014
**21**	2,5,6,7-Tetramethoxyflavan	6.15	2.67	C_19_H_22_O_5_	[M + H]^+^	331.1541	331.1540	0.30	328.0938, 313.0702, 287.0554, 231.0654, 131.0491, 91.0538
**22**	Calycinine	6.24	1.76	C_18_H_17_NO_4_	[M + H]^+^	312.1229	312.1230	−0.32	295.0963,294.1123, 0858, 265.0858, 237.0912, 236.4374
**23**	Oxodiscoguattine	6.31	2.13	C_19_H_13_NO_5_	[M + H]^+^	336.0856	336.0866	−2.98	336.0862, 321.0627, 318.0764,263.0581, 246.0548, 178.0657
**24**	noraristolodione	6.43	1.18	C_17_H_11_NO_4_	[M − H]^−^	292.0620	292.0615	1.61	278.0415, 277.0382, 250.0463, 249.0432, 221.0481, 140.4708, 91.9955
**25**	Asimilobine	6.69	2.38	C_17_H_17_NO_2_	[M + H]^+^	268.1329	268.1332	−1.12	252.1100, 251.1065, 236.0830, 219.0803, 191.0855, 103.6783
**26**	Aristolactam AII	6.71	1.24	C_16_H_11_NO_3_	[M + H]^+^	266.0809	266.0812	−1.13	251.0581, 249.0433, 238.0861, 223.0628, 221.0482, 195.0679
**27**	Glaucine	6.89	2.34	C_21_H_25_NO_4_	[M + H]^+^	356.1852	356.1856	−1.12	325.1431, 310.1196, 295.1282, 294.1248, 251.1062
**28**	Aloenin	6.91	1.36	C_19_H_22_O_10_	[M − H]^−^	409.1140	409.1140	0.00	229.0354, 214.0121, 170.9937, 142.9987, 111.0087, 83.0138
**29**	*N*-Jasmonoyl-L-isoleucine	7.12	2.12	C_18_H_29_NO_4_	[M + H]^+^	322.2025	322.2024	0.37	322.2026, 271.0607, 165.1286, 130.0874, 128.1082, 58.0299
**30**	Fissistigmatin A	7.31	2.36	C_33_H_42_O_5_	[M + H]^+^	519.3104	519.3105	−0.19	519.3105, 501.2999, 487.2843, 469.2737
**31**	Duguevanine	7.31	2.28	C_20_H_21_NO_5_	[M + H]^+^	356.1491	356.1492	−0.28	325.1068, 310.1197, 294.1249, 267.1013, 255.1013, 81.4017
**32**	Licocoumarin A	7.34	2.28	C_25_H_26_O_5_	[M + H]^+^	405.1708	405.1707	0.13	343.1705, 293.1143, 292.1108, 274.1003, 256.1107, 201.0058, 157.0657
**33**	Globulixanthone A	7.38	3.15	C_19_H_16_O_5_	[M + H]^+^	325.1063	325.1071	−2.46	296.0995, 295.0963, 267.1016, 265.0856, 253.0858, 225.0907
**34**	Clausamine F	7.78	3.41	C_19_H_19_NO_4_	[M + H]^+^	326.1382	326.1387	−1.53	310.1152, 309.1119, 294.0884, 279.1014, 265.0862, 251.1061, 248.0830
**35**	**Crebanine**	7.87	2.19	C_20_H_21_NO_4_	[M + H]^+^	340.1546	340.1543	0.88	309.1118, 308.1062, 294.0884, 279.1013, 239.1063, 236.0833
**36**	Hippeastrine	7.96	2.67	C_17_H_17_NO_5_	[M + H]^+^	314.1036	314.1034	0.65	299.0802, 284.0568, 267.0540, 255.0302, 208.0252, 179.9940
**37**	Ammidin	8.44	2.26	C_16_H_14_O_4_	[M − H]^−^	269.0818	269.0819	−0.37	269.0820, 254.0586, 226.0636, 171.0451, 165.0191, 122.0008
**38**	Thunberginol C	8.58	1.51	C_15_H_12_O_5_	[M + H]^+^	271.0614	271.0612	0.75	271.0615, 227.0716, 151.0037, 119.0502, 107.0138, 93.0345
**39**	[3-3″]bi-2-hydroxy-4,5,6-trimethoxydihydrochalcone	8.59	1.35	C_36_H_38_O_10_	[M + H]^+^	631.2534	631.2538	−0.63	631.2538, 615.2589, 599.2639, 467.1700
**40**	5, 6, 7, 8-tetramethoxyflavone	8.66	1.72	C_19_H_18_O_6_	[M + H]^+^	343.1174	343.1176	−0.63	343.1172, 328.0938, 314.0737, 313.0702, 282.0884
**41**	1, 2-Dihydrotan-shinquinone	8.68	3.42	C_18_H_14_O_3_	[M + H]^+^	279.1011	279.1016	−1.79	279.1012, 249.0907, 221.0958, 206.0728, 178.0776
**42**	Oxocrebanine	8.69	3.51	C_19_H_13_NO_5_	[M + H]^+^	336.0865	336.0866	−0.30	336.0864, 321.0629, 319.0793, 306.0406, 278.0453, 250.0503
43 *	CAS 1391982-39-0	8.71	1.69	C_17_H_18_O_5_	[M + H]^+^	303.1221	303.1227	−1.98	285.1126, 253.0861, 225.0912, 169.0496, 105.0700, 91.0543
**44**	Anolobine	8.87	2.22	C_17_H_15_NO_3_	[M + H]^+^	282.1121	282.1125	−1.42	265.0855, 235.0750, 209.0957, 207.0802, 121.2282
**45**	Procyanidin B	9.148	2.90	C_30_H_26_O_12_	[M + H]^+^	577.1347	577.1351	−0.78	407.0771, 289.0719, 245.0819, 161.0243, 137.0243, 125.0243
**46**	Isolaureline	9.51	3.65	C_19_H_19_NO_3_	[M + H]^+^	310.1437	310.1438	−0.32	310.1432, 280.1047, 279.1014, 249.0908, 221.0957
**47**	Xylopine	9.56	1.51	C_18_H_17_NO_3_	[M + H]^+^	296.1278	296.1281	−1.01	280.1041, 279.1014, 249.0912, 240.0747, 102.7522, 95.9003
**48**	Fissilandione	9.6	3.14	C_19_H_15_NO_5_	[M + H]^+^	338.1019	338.1023	−1.18	338.1020, 308.0918, 280.1045, 279.1014, 249.0908, 221.0961, 178.0770, 92.0010
**49**	Daphmanidin E	10.65	2.11	C_25_H_31_NO_5_	[M + H]^+^	426.2273	426.2275	−0.47	426.2274, 410.2325, 408.2167, 392.2223
**50**	Fissistigmatin C	11.34	1.38	C_33_H_40_O_4_	[M + H]^+^	501.2996	501.2999	−0.60	501.2996, 473.2684, 469.2732, 365.1751
**51**	Atherospermidine	12.52	2.04	C_18_H_11_NO_4_	[M + H]^+^	306.0753	306.0761	−2.61	306.0758, 307.0791, 291.0523, 278.0815, 263.0575,76.3926
**52**	Fissistigine C	14.42	1.24	C_20_H_23_NO_4_	[M + H]^+^	342.1695	342.1700	−1.46	342.1697, 311.1278, 296.1043,285.1120, 280.1092, 191.0940, 162.0915, 58.0658
**53**	Byakangelicin	15.93	2.42	C_17_H_18_O_7_	[M − H]^−^	333.0983	333.0980	0.97	289.1085, 258.0854, 257.0821, 205.0871, 173.0607, 125.0243, 101.0243, 92.9981
**54**	Aristolactam BIII	17.07	2.10	C_18_H_15_NO_4_	[M + H]^+^	310.1075	310.1074	0.32	310.1072, 295.0838, 280.0603, 277.0732, 248.0702, 98.5990

The standards used in this experiment were marked in bolds; 43*: CAS number of 8,9-dimethoxy-7-met-hyl-10,11-dihydrodib-enzo[b,f]oxepine-1,6-diol.

**Table 2:** Different compounds in five parts of *F. oldhamii.*

No.	Name	RT	Formula	Ion Species	Observed *m*/*z*	Calculated *m*/*z*	Diff. (ppm)	MS/MS Fragments	GEN	JING	YE	GUO	CY
**55**	Artabotryside A	4.35	C_26_H_28_O_15_	[M − H]^−^	579.1359	579.1355	0.62	579.1361, 301.0314, 300.0277, 271.0250, 255.0301, 178.9986, 151.0035	-	✓	✓	✓	✓
**56**	Apiin	4.76	C_26_H_28_O_14_	[M − H]^−^	563.1410	563.1406	0.66	563.1411, 503.1199, 473.1091, 443.0988, 383.0775, 354.0703, 353.0669	-	✓	✓	✓	✓
**57**	Nicotiflorin	4.90	C_27_H_30_O_15_	[M − H]^−^	593.1511	593.1512	−0.16	593.1514, 503.1198, 473.1090, 383.0773, 353.0667	-	✓	✓	✓	✓
**58**	Eupatolin	4.94	C_23_H_24_O_12_	[M − H]^−^	491.1198	491.1195	0.61	491.1200, 330.0703, 329.0656, 328.0590, 314.0424, 313.0357,282.0535	-	✓	✓	✓	✓
**59**	Aristolactam FII	5.59	C_17_H_13_NO_4_	[M − H]^−^	294.0776	294.0772	1.42	294.0779, 280.0572, 279.0539, 265.0338, 264.0304	✓	✓	-	✓	✓
**60**	Quercetin-3-*O*-rhamn-oside	6.33	C_21_H_20_O_11_	[M − H]^−^	447.0937	447.0933	0.93	447.0934, 358.0652, 357.0618, 328.0547, 327.0513, 299.0562, 285.0408	-	✓	✓	✓	✓
**61**	**Aristolactam FI**	6.80	C_16_H_11_NO_3_	[M + H]^+^	266.0810	266.0812	−0.64	266.0807, 251.0582, 249.0909, 219.0803, 191.0857,84.4612	✓	✓	-	-	✓
**62**	Cnidimol B	7.04	C_15_H_16_O_6_	[M − H]^−^	291.0878	291.0874	1.33	219.1027, 188.0798, 187.0765, 172.0530, 155.0503, 145.0659, 83.0139	-	-	✓	✓	-
**63**	Isoquercitrin	7.33	C_21_H_20_O_12_	[M − H]^−^	463.0888	463.0882	1.30	463.0886, 301.0341, 300.0277, 271.0251, 255.1028, 178.9986, 151.0036	-	-	✓	-	-
**64**	Stigmahamone II	7.45	C_18_H_18_O_7_	[M − H]^−^	345.0982	345.0980	0.58	330.0747, 315.0513, 298.0483, 283.0249, 255.0300, 239.0198, 200.0717	✓	-	-	✓	-
**65**	Claussequinone	7.81	C_16_H_14_O_5_	[M + H]^+^	287.0908	287.0914	−2.09	287.0910, 271.0966, 257.0819, 183.0291,149.0591, 131.0492, 91.0542	✓	-	✓	✓	-
**66**	Oxoglaucine	8.40	C_20_H_17_NO_5_	[M − H]^−^	350.1034	350.1034	0.01	335.0801,307.0852, 279.0902, 231.0173, 231.0173, 203.0226, 175.0274	✓	✓	✓	-	✓
**67**	Methyl 3-(2-oxo- 2-prop-2-enoxyethyl)-1-benzofuran-2-carboxylate	8.64	C_15_H_14_O_5_	[M + H]^+^	273.0772	273.0768	1.29	259.0568, 258.0534, 168.0018, 166.9985, 139.0037	✓	-	✓	✓	-
**68**	Morusin	8.73	C_25_H_24_O_6_	[M − H]^−^	419.1501	419.1500	0.24	386.1161, 276.1635, 375.1602, 357.1498, 270.0855, 269.0821	-	-	✓	✓	-
**69**	Kwangsienin A	8.77	C_18_H_18_O_6_	[M + H]^+^	331.1168	331.1176	−2.42	230.0416, 228.0580, 227.0546, 212.0312, 197.0079, 169.0142, 139.0389, 85.0285	✓	✓	-	✓	✓
**70**	**Isopedicin**	9.44	C_18_H_18_O_6_	[M + H]^+^	331.1172	331.1176	−1.25	316.0949, 227.0557, 212.0320, 197.0084, 169.0136, 113.0233, 85.0280	✓	✓	-	✓	✓
**71**	Kanakugiol	9.52	C_19_H_20_O_6_	[M + H]^+^	345.1330	345.1333	−0.77	242.0736, 241.0702, 226.0468, 211.0235, 183.0287, 131.0490	✓	-	-	✓	-
**72**	Rottlerin	9.91	C_30_H_28_O_8_	[M + H]^+^	517.1853	517.1857	−0.77	499.1742, 485.1605, 468.2096, 385.1286, 231.1024, 184.0736, 105.0700, 91.0541	✓	-	✓	✓	✓
**73**	Nomilin	9.96	C_28_H_34_O_9_	[M − H]^−^	513.2126	513.2130	−0.79	481.1867, 453.1920, 307.1704, 289.0717, 178.9985, 151.0399,123.0450	✓	-	-	✓	-
**74**	Proceranolide	10.59	C_27_H_34_O_7_	[M + H]^+^	471.2376	471.2377	−0.21	403.1756, 353.1379, 297.0758, 261.1484, 233.0807, 221.0807, 201.0544, 193.0858	-	-	✓	✓	✓
**75**	Aristolactam BII	12.35	C_17_H_13_NO_3_	[M + H]^+^	280.0965	280.0968	−1.14	280.0965, 265.0732, 264.0652, 240.0629, 236.0703, 149.0234	✓	✓	-	-	✓
**76**	Fissohamione	12.72	C_16_H_18_O_5_	[M + H]^+^	291.1226	291.1227	−0.34	273.1904, 141.0574, 105.0721	-	-	✓	✓	-
**77**	Norfissilandione	17.78	C_18_H_13_NO_5_	[M + H]^+^	324.0866	324.0866	−0.15	324.0864, 312.1001, 309.1121, 295.0960, 266.0810	✓	-	-	-	-
**78**	Norcepharadione B	19.83	C_18_H_13_NO_4_	[M + H]^+^	308.0910	308.0917	−2.27	308.0911, 293.0678, 279.0839, 250.0856, 156.3622	✓	-	-	-	-
**79**	Gedunin	19.88	C_28_H_34_O_7_	[M − H]^−^	481.2235	481.2232	0.67	449.1976, 434.1734, 419.1509, 401.1396, 391.1556, 373.1449, 198.0172, 182.9936, 152.9830	✓	-	-	-	-

The standards used in this experiment were marked in bolds; “✓” implied the compounds in the related part, while “-” meat no distribution.

**Table 3:** Method validation parameters of eight detected standards.

Standards	Precision (RSD, *n* = 6)		Repeatability	Stability	Regression Equation (*n* = 3)	Linear Range *	R^2^	LOQ *	LOD *	Added (ng)	Detected (ng)	Recovery (%)	RSD (%) *n* = 3
	Intra-day	Inter-day	(RSD, *n* = 6)										
Syringic acid	2.2	4.6	3.8	2.8	y = 0.688x + 0.0147	0.03–4	0.9994	3.2	1	100	99.5 ± 0.5	99.5	0.50
(−)-Epicatechin	3.2	4.4	2.3	3.3	y = 0.10x − 0.0022	0.03–1	0.9997	2.2	0.5	50	51.75 ± 0.72	103.5	1.39
Crebanine	2.9	3.1	3.3	3.2	y = 0.773x − 0.010	0.06–4	0.9999	0.7	0.2	25	25.17 ± 0.90	100.68	3.58
Corytuberine	3.8	4.3	2.9	4.6	y = 0.318x + 0.004	0.06–1	0.9991	1.3	0.4	50	50.9 ± 0.68	101.8	1.34
Isopedicin	2.7	4.5	3.2	2.6	y = 0.500x − 0.083	0.03–4	0.9999	1.5	0.4	50	52.0 ± 0.91	104	1.75
Aristolactam BII	1.1	4	2.8	2.8	y = 0.172x − 0.007	0.125–4	0.9988	2.2	0.5	100	100.2 ± 0.93	100.2	0.93
Aristolactam FI	2.3	1.8	3.2	3.2	y = 0.720x − 0.026	0.125–1	0.9991	0.8	0.2	50	50.6 ± 0.54	101.2	1.07
Aristolactam AIIIa	4.8	4.9	4.5	4.8	y = 0.793x − 0.029	0.06–4	0.9992	0.1	0.03	50	49.9 ± 0.38	99.8	0.76

* The unit of linear range is μg·mL^−1^, while LOD and LOQ was in ng·mL^−1^.

## Data Availability

The data presented in this study are available on request from the corresponding authors.

## Sample Availability

Not available.
